# The plant endophytic fungus *Cyanodermella asteris* produces the phytohormone jasmonic acid

**DOI:** 10.1186/s40694-026-00210-6

**Published:** 2026-04-03

**Authors:** Linda Jahn, Angela Sester, Elena Theresa Domaschke, Tobias A. M. Gulder, Jutta Ludwig-Müller

**Affiliations:** 1 Plant Physiology, Faculty of Biology, TU Dresden, 01062 Dresden, Germany; 2 Faculty of Medicine Carl Gustav Carus, Else Kröner Fresenius Center for Digital Health, TU DresdenFetscherstraße 74, 01307 Dresden, Germany; 3 Faculty of Chemistry, Chair of Technical Biochemistry, TU Dresden, 01062 Dresden, Germany; 4 Department of Natural Product Biotechnology, Helmholtz Institute for Pharmaceutical Research Saarland (HIPS), Helmholtz Centre for Infection Research (HZI) and Department of Pharmacy at Saarland University, PharmaScienceHub (PSH), 66123 Saarbrücken, Germany

**Keywords:** *Arabidopsis thaliana*, *Cyanodermella asteris*, Jasmonic acid, Endophyte, Plant stress response

## Abstract

**Background:**

*Cyanodermella asteris* is a fungal endophyte from *Aster tataricus* that produces plant hormones as well as a range of specialized metabolites. The aim of our study was to explore the potential of this endophytic fungus towards plant hormones besides the auxin indole-3-acetic acid which we recently identified.

**Results:**

Here, we identified another hormone, jasmonic acid (JA), from culture medium extracts by LC-MS/MS and NMR. JA was also found in the hyphal fraction, but its *de novo* biosynthesis could not be stimulated by linolenic acid, a known precursor for JA biosynthesis in plants. The growth of *C. asteris* in media was not inhibited by JA. Only at high concentrations of 1 mM, an inhibition of biomass production was recorded. Putative genes encoding enzymes for JA biosynthesis were identified in the genome, and expression analyses showed an induction of one thioester hydrolase, possibly catalyzing saponification of JA-CoA to free JA. We also investigated its interaction with plant jasmonate biosynthesis and signaling mutants, *aoc* and *jar*, respectively, and found that the fungus can complement the JA-deficient phenotypes.

**Conclusions:**

Further understanding of the biology of JA biosynthesis on *C. asteris* as well as its interactions with plants is needed to exploit its potential use as a producer of JA.

**Supplementary Information:**

The online version contains supplementary material available at 10.1186/s40694-026-00210-6.

## Background

Jasmonic acid (JA), a fatty acid and oxylipin derivative [10], is best known in plants, where it plays a critical role as a phytohormone in plant defense, growth, and development [21]. Pathogen attack by fungi and bacteria activates JA production and initiates the immune response in plants [3, 37]. JA is activated by mechanical damage like wounding through herbivores or environmental stress (reviewed in [66]). JA helps plants cope with abiotic stresses such as drought, heat or salinity by modulating stomatal closure or metabolic pathways (reviewed in [63]). JA can alter flowering time and seed development, among other things, in response to environmental stress. Together with other hormones, JA plays a role in fruit ripening and senescence as well as root growth [64]. These are just a few examples of how JA interacts with other phytohormones to trigger plant growth and development as well as the plant interaction with its biotic and abiotic environment.

Besides higher plants, JA is also known from cyanobacteria such as *Spirulina* [58], algae such as *Chlorella*, *Gelidium* or *Scenedesmus* [9, 32, 58] and some phytopathogenic fungi such as *Botryodiplodia theobromae* [34], *Lasiodiplodia theobromae* [12], *Fusarium oxysporum* [40] or *F. fujikuroi* [12]. The function of JA in fungi is still not well understood. So far, fungal JA is thought to influence the growth and stress response of host plants [14], while in the fungus *Magnaporthe oryzae*, JA induces a switch from vegetative to pathogenic growth and to the formation of infectious structures [35]. Fungal jasmonates could either induce [7] or attenuate the plant defense responses [45] depending on the respective infection strategy, while plants themselves also react with induction of the jasmonate pathway to infection by necrotrophic fungi [37].

JA biosynthesis in plants is a complex process (for an overview on JA biosynthesis, cf. Figure ) [12, 65, 66, 67]. It starts with the release of fatty acids from plastid membranes, usually from linolenic acid (LA), which is known to be part of these membranes. LA is then oxygenated by a lipoxygenase (LOX) to the key intermediate 13-hydroperoxylinolenic acid (13(*S*)-HPOT). The next reactions are carried out by an allene oxide synthase (AOS) and an allene oxide cyclase (AOC) to produce the intermediate 12-oxophytodienoic acid (OPDA). OPDA is finally reduced to JA by several enzymes. In plants, the major steps of JA biosynthesis take place in the plastids. Since fungi do not have such organelles, the pathway has to be present in different compartments and might also involve different enzymes. *Lasiodiplodia theobromae* also uses LA as a substrate for JA biosynthesis [12]. *Fusarium oxysporum* contains the intermediates OPDA and 13(*S*)-HPOT [43]. Additionally, enzymes for the catalysis of at least the possible primary step, like lipoxygenases, have now been reported from fungi as well [42].

The endophytic fungus *Cyanodermella asteris* was isolated from the perennial plant *Aster tataricus* [27], long known in Traditional Chinese Medicine for its antibacterial, antiviral, antiulcer, and diuretic effects [52, 53, 54]. *C. asteris* was found to synthesize secondary metabolites such as the polyketide skyrin [27] and the astin family of pentapeptides [51, 60]. It also produces the phytohormone indole-3-acetic acid (IAA) [26], which can be taken up by the non-host plant *Arabidopsis thaliana*.

To better understand the interactions with (host) plants, we analyzed the culture medium of *C. asteris* for other phytohormones known to play a role in plant-endophyte interactions. Intensive analysis of the supernatant led to the isolation of JA, and its identification by mass spectrometry and NMR, reporting it for the first time for this fungal family. Several questions arose from that finding, including the biosynthetic origin of JA and how much of JA is secreted into the medium. By feeding *C. asteris* cultures with the plant JA precursor LA, we evaluated if *C. asteris* utilizes LA to produce JA. The effect of exogenous JA on biomass and astin C production in *C. asteris* was also assessed. Co-cultivation assays with the model plant *A. thaliana* were executed to show whether fungal JA could be taken up by plants. Here, we used the JA biosynthesis-deficient mutant *A. thaliana aos10-1*, which exhibits a JA-deficient phenotype with delayed flowering and seed development.

## Materials and methods

### Biological material

*C. asteris* strain 03HOR08 was isolated from the plant *A. tataricus*, obtained from Sarastro Stauden in Austria, as described earlier [27] (deposited under the accession number DSM 100826 at DSMZ, Braunschweig, Germany). The *A. thaliana* ecotype Columbia (Col-0, N1092) and the JA-signaling mutant *jar1-1* (N8072) were obtained from the Nottingham Arabidopsis Stock Centre (NASC, Loughborough, U.K.). The JA biosynthesis-deficient mutant of *A. thaliana aos10-1* was a gift from Stephan Pollmann (Universidad Politécnica de Madrid, Madrid, Spain).

### Cultivation of *C. asteris*

For identification of JA, *C. asteris* was cultivated in a 180 ml pre-culture using MEAlow medium (10 g/l malt extract, 10 g/l D-glucose, 1 g/l peptone, 1 ml/l Hutner’s trace elements according to Hutner et al., [24], pH 6.1 ± 0.1) for one week. The pre-culture was centrifuged (5,000 rcf, 10 min, 20 °C) and the pelleted hyphae were resuspended in fresh MEAlow medium (10 ml fresh medium per 1 ml pellet). The resuspended pellet was homogenized using a glas homogenizer. The main cultures were set up in six 3 l Fernbach flasks, each containing 2 l MEAlow and inoculated with 20 ml *C. asteris* homogenate. They were cultivated at 22 °C at 130 rcf for three months in the dark.

### Cultivation of *C. asteris* with JA

*C. asteris* was cultivated in 20 ml MEAlow (minimal medium) and 20 ml MEA3 (full medium (20 g/l malt extract (Oxoid), 10 g/l D-glucose, 30 g/l mannose, 1 g/l peptone (from meat), 1 ml/l Hutner’s trace elements [24], pH = 6.1 ± 0.1) for four weeks in the presence of different concentrations of (±)-JA (ThermoFisher Scientific, Waltham, MA/USA). The cultures were inoculated with 250 µl *C. asteris* homogenate as described above and were cultivated at 130 rcf and 23 °C in the dark. After one week, JA was added to the *C. asteris* cultures, giving final concentrations of 1 nM to 1 mM. JA was dissolved in ethanol and the ethanol concentration was kept below 0.1% in the cultures. The cultures were harvested by centrifugation (5.000 rcf, 10 min, 4–8 °C), and biomass was determined after freeze drying as described earlier in [28].

### Cultivation of *C. asteris* with LA

For feeding experiments with LA (Carl Roth GmbH & Co. KG, Karlsruhe, Germany), *C. asteris* was cultivated in Erlenmeyer flasks containing 50 ml MEAlow and different amounts of LA. Each culture was inoculated with 250 µl homogenate from a one-week-old pre-culture of *C. asteris*. LA was dissolved either in DMSO (final concentrations of 0.1/0.2/0.5/0.7/1/2/5 mM), or methanol (0.1/0.5/1/2/5 mM). The highest amount of solvent per flask was 0.1% to ensure a non-inhibited growth of *C. asteris* by the different solvents. The flasks were cultivated on a shaking incubator at 23 °C, 130 rcf, in the dark for four and eight weeks (methanol: four and eight weeks; DMSO: eight weeks).

### Co-cultivation of *C. asteris* with *A. thaliana*

*C. asteris* was co-cultivated with the plant *A. thaliana* as described earlier [26]. In short, plates containing ½ MS/MEAlow (1.1 g/l Murashige and Skoog medium incl. vitamins, 5 g/l malt extract, 5 g/l D-glucose, 0,5 g/l peptone, 0,5 ml/l Hutner’s trace elements [24], pH 6.1 ± 0.1) were inoculated with sterile seeds of *A. thaliana*, either wild type Col-0, JA-mutants *aos10-1* (JA deficient) or *jar1-1* (JA signaling). The seeds were sterilized using 70% ethanol with 0.1% Triton X-100 for 1 min and 1.2% sodium hypochlorite with 0.1% Triton X-100 for 1 min followed by washing the sterile seeds three times with autoclaved water. The seeds were shaken during the sterilization process. The sterile seeds were stratified at 8 °C in the dark for 2 d. Sterile, stratified seeds were transferred to the upper part of the co-cultivation plates (seven seeds per plate), which were sealed with Parafilm and cultivated for one week under long day conditions in the climate chamber (23 °C for 16 h with light, 18 °C for 8 h in the dark) in an upright position. After one week, the plates were opened under sterile conditions and 5 µl *C. asteris* homogenate from a one-week-old culture was added under each seedling in a distance of 3.5 cm. The plates were sealed with MaiMed tape (MaiMed GmbH, Neuenkirchen, Germany) to ensure gas exchange in an upright position for another four weeks. Controls without *C. asteris* were treated in the same way without adding homogenate to the plates (ten plates per treatment). Root length, rosette diameter as well as growth stage of the plants and fungal diameter were measured each week. After a total of five weeks, the plants were harvested and fresh as well as dry weight of the roots and rosettes, including inflorescences, were determined separately. The growth stages of the differently treated plants were statistically analyzed in each week using a Kruskal-Wallis-ANOVA, followed by a Dunn’s test [11, 33].

### Isolation and identification of JA via LC-MS and NMR

*C. asteris* cultures were harvested by centrifugation (5000 rcf, 10–15 min, 20 °C) to separate medium and hyphae. For identification of JA, the 12 l medium (supernatant) was analyzed. The supernatant was extracted once in a separating funnel with the same volume of ethyl acetate. The organic phase was dried over magnesium sulfate, filtered, and then dried under reduced pressure using a rotary evaporator. The raw extract was dissolved in 5 ml acetonitrile and submitted to HPLC purification.

The raw extract was separated over a preparative HPLC using an UV/Vis detector at 240 nm coupled with a Foxy R1 Fraction Collector (Knauer Wissenschaftliche Geräte GmbH, Berlin, Germany). Separation was achieved on an Eurospher II 100-5 C18A column 250 × 16 mm (Knauer Wissenschaftliche Geräte GmbH) using a gradient of water and acetonitrile both incl. 0.05% trifluoroacetic acid. The gradient of acetonitrile was increased over 45 min from 5% to 98% and kept at 98% for 8 min at a constant flow rate of 10 ml/min. Fractions were collected with time frames of 1 min. Data analysis was performed with the software ClarityChrom (Knauer Wissenschaftliche Geräte GmbH).

Selected fractions were dried and dissolved in 2 × 500 µl acetonitrile. To remove residual water, the resulting samples were vacuum-centrifuged, and freeze-dried, followed by NMR and MS analyses.

For NMR measurements, samples were dissolved in 700 µl CD_3_OD. ^1^H and ^13^C Nuclear Magnetic Resonance spectra (NMR) were recorded on a Bruker AVANCE 600 spectrometer at room temperature. The chemical shifts are given in δ-values (ppm) downfield from TMS and are referenced on the residual peak of the deuterated solvent (CD_3_OD: δ_H_ = 3.31 ppm, δ_C_ = 49.0 ppm). The coupling constants are given in Hertz [Hz]. Spectra were processed with the Bruker TopSpin 4.1.4 Software and ACD/NMR Workbook 2021.2.0. ^13^C spectra were measured with proton decoupling (^13^C {^1^H}).

For UHPLC-HRMS analysis, a Vanquish UPLC device manufactured by Thermo Scientific, coupled with a Bruker ESI-QqTOF impact II mass spectrometer was used. The HPLC consists of the following components: VF-D11-A diode array detector FG, VF-A10-A split sampler FT, VF-P10-A binary pump, column compartment. The system was controlled by Bruker Hystar and otof Control software.

LC conditions and couplings were as follows: An Acquity UPLC BEH C18 column (130 Å, 1.7 μm, 100 × 2.1 mm) with an Acquity UPLC BEH C18 VanGuard pre-column (130 Å, 1.7 μm, 5 × 2.1 mm) and KrudKatcher Ultra HPLC in-line filter (2.0 μm depth filter x 0.004 in ID) – all by Waters Corporation, Milford, Massachusetts (US). It was used with the following eluent solvent: A = H_2_O + 0.1% formic acid, B = MeCN + 0.1% formic acid at 45 °C column oven temperature. The separation method consisted of the following gradient system: 0–0.5 min 15% B, 0.5–18.5 min to 30% B, 18.5–19.5 min to 95% B 19.5–20.5 min 95% B, 20.5–20.8 min to 5% B, 20.8–22.5 min 5% B with a flow rate of 0.6 ml/min. The LC flow was split to 75 µl/min before it was forwarded to the MS unit.

*MS only settings*: Parameters for the Bruker impact II were as follows: Mass spectra were acquired in the centroid (line) and profile mode ranging from 50 to 1300 *m/z* at a 12 Hz full scan rate. Mass spectrometry source parameters were set to a 500 V end plate offset, a 4500 V capillary voltage, a 0.6 bar nebulizer gas pressure, a 5.1 l/min dry gas flow, and a 200 °C dry gas temperature. Ion transfer and quadrupole settings were set to funnel 1 RF 200 Vpp.; funnel 2 RF 200 Vpp as transfer settings and ion energy of 5 eV as well as a low mass cut of 50 *m/z*.

#### MS/MS settings

For MS/MS experiments were carried out in MRM mode at 2 Hz spectra rate, selecting for mass 211.1329 and 233.1148 (corresponding to JA *m/z* [M + H]^+^ and [M + Na]^+^, respectively), with width 1 and 5, isCID 0.00 and collision energy 10–50 eV. Data analysis was performed with Bruker Data Analysis 6.1 software. (±)-jasmonic acid analytical reference was purchased from Sigma Aldrich (J2500-100MG, CAS 77026-92-7).

### Quantification of JA in medium and hyphae

For feeding experiments, both medium and hyphae were analyzed. After separation of medium and hyphae by centrifugation, the medium was extracted twice with the same volume of ethyl acetate after adding 10 µl of 1 mg/ml internal standard (±)-D_5_-JA (Cayman Chemical, Ann Arbor, MI/US) for quantification. The internal standard and the ethyl acetate were added at the same time to the samples. These were vortexed and centrifuged (5,000 rcf, 10 min, 20 °C). The upper organic phase was collected and the lower aqueous phase was again extracted with the same volume of ethyl acetate. Both organic phases were combined, vacuum-dried and resolved in 5 ml 0.1 M acetic acid. The extract was cleaned over a SPE C18 column (Chromabond C18 ec, 45 μm, 6 ml/500 mg, from Macherey Nagel, Düren, Germany; P/N 730014) using a compressor. The SPE column was equilibrated with 5 ml 50% methanol in 0.1 M acetic acid. The extract was added onto the column and the flow-through was discarded. The SPE column was washed once with 5 ml 40% methanol in 0.1 M acetic acid, before JA was eluted from the SPE column by 5 ml 50% methanol in 0.1 M acetic acid. The extract containing JA was dried and dissolved in 1 ml of 100% methanol. To remove residues from previously used solvents, the extract was additionally dried under a nitrogen stream. The final extract was dissolved in 100 µl methanol and kept in the freezer at -20 °C until LC-MS measurements.

The hyphae (pellet) were washed three times with water to remove residual medium and then freeze-dried. Freeze-dried pellets were ground and extracted with 50 ml of 70% methanol including 10 µl of 1 mg/ml internal standard (±)-D_5_-JA on a shaker in the dark at 4–8 °C for 30 min. The extract was centrifuged (5,000 rcf, 10 min, 20 °C) to separate undissolved biomass from the supernatant. In the latter, the organic solvent was removed under reduced pressure, leaving the aqueous extract. This was extracted twice with the same volume of ethyl acetate and further handled as described above for the medium.

For quantification, the samples were analyzed using a QExactive Plus Orbitrap LC-MS/MS system (Thermo Fisher Scientific, Waltham, MA/USA) equipped with an Accucore Vanquish C18 + column (50 × 2.1 mm, 1.5 μm particle size; Thermo Fisher Scientific). Chromatographic separation was performed using a gradient elution starting from 5% acetonitrile (ACN) in water, each supplemented with 0.06% (in ACN) and 0.1% acetic acid (in water). The gradient increased to 60% ACN over 15 min, followed by a rise to 98% ACN over the next 3 min. This was maintained for an isocratic phase of 5 min at 98% ACN, before re-equilibrating to 5% ACN within 2 min. The flow rate was set to 0.25 ml/min at a column temperature of 30 °C. Ionization was carried out via electrospray ionization (ESI) using the Thermo Fisher Scientific Ion Max API source, samples were measured in negative mode. Chromatograms and mass spectra were analyzed using Freestyle 1.7 software (Thermo Fisher Scientific).JA was quantified using the internal standard (±)-D_5_-JA on the isotope dilution equation [25], following equations (I + II):

1 $$\:m\left(JA\right)\left[\mu\:g\right]=\left(\frac{area\left(209\right)+area\:\left(212\right)}{area\:\left(212\right)}-1\right)\times\:m\left(D5JA\right)\left[\mu\:g\right]$$

2 $$\:c\left(JA\right)=\:\frac{m\left(JA\right)\left[\mu\:g\right]}{dry\:weight\:hyphae\:\left[mg\right]}$$

Therein, area (209) represents the EIC peak area under the curve of unlabeled JA, area (212) stands for EIC peak area under the curve of heavy labeled (±)-D_5_-JA, and m(D5JA) equals the amount of supplemented (±)-D_5_-JA during extraction. The relative JA secretion of *C. asteris* into the medium was expressed as the percentage of JA in the medium relative to the total JA (medium + hyphae).

### RNA extraction and qPCR analysis

Two samples of *C. asteris* were chosen for analysis of JA biosynthesis gene expression: a four-week-old culture with less JA production (approx. 5 µg/g dry weight) and an eight-week-old culture with higher JA production (approx. 80 µg/g dry weight), both controls from the feeding experiments with LA. Freeze-dried hyphae of these two *C. asteris* cultures were used to isolate RNA with RNAzol (Sigma Aldrich, St. Louis, MO, USA). The isolated RNA was digested with DNAse I (Thermo Fisher Scientific, Waltham, MA, USA) to remove residual DNA, and concentrated using RNA Clean & Concentrator Kit (Zymo Research Europe GmbH, Freiburg, Germany). The quantity and quality of the resulting DNA-digested RNA were analyzed using a NanoDrop™ ND1000 (PeqLab Biotechnologie GmbH, Erlangen, Germany) and a 2100 Bioanalyzer (Agilent, Santa Clara, CA, USA) with an Agilent RNA 6000 Nano kit. The cDNA was synthesized using the Maxima First Strand cDNA Synthesis kit (Thermo Fisher Scientific, Waltham, MA, USA).

Due to the large number of potential candidate genes of the JA biosynthesis after a KEGG analysis [30] of the genome, only a few were selected. Genes with peroxisome signals were chosen as OPDA activation and β-oxidation occur in peroxisomes [12]. Genes that were assigned to a biosynthetic gene cluster by automatic annotation using GenDBE were excluded, since it is not known whether genes involved in JA biosynthesis are organized in clusters. qPCR with four candidate genes of the JA biosynthesis pathway was conducted: three encoding putative thiolester hydrolases *(3398_g*, *8240_g* and *8653_g*), and one putative acyl CoA oxidase gene (*2369_g*). Specific primers (Suppl. Table 1) were used to amplify reference genes (coding for γ-actin gACT, elongation factor 1-beta EF1b, DNA-directed RNA polymerase II subunit RPB2) and the selected genes of interest (GOIs) (*3398_g*,* 8240_g*,* 8653_g*,* 2369_g*) for expression analysis. qPCR was done with a qTOWER 2.2 (Analytik Jena, Jena, Germany), using the qPCRBIO SyGreen Mix Separate-ROX (Nippon Genetics Europe, Dueren, Germany). The qPCR program was as follows: 95 °C for 2 min, followed by 40 cycles of 95 °C for 5 s and 64 °C for 25 s, concluding with a melting curve at the end.

Since the standard deviations between the technical replicates of each run were below a Ct-value of 0.5, the mean of the nine technical replicates of each run was used to calculate the expression levels with the Relative Expression Software Tool Multiple Condition Solver [46]. A log2 ratio of 1 is considered as up-regulation, a log2 ratio of -1 as down-regulation of the respective gene.

## Results

### Identification of JA from *C. asteris*

Screening of the organic extract from a 12 l *C. asteris* liquid culture for secondary metabolites and phytohormones was performed by compound purification by preparative HPLC and subsequent analysis of the fractions by LC-HR-MS and NMR. One fraction stood out by its distinct NMR pattern and extensive 1D and 2D NMR experiments led to the identification of JA as the main compound (Suppl. Figure 1).

From the NMR, the relative stereochemical configuration could be interpreted as a *(Z)-*configured pentenyl side chain and a 1,2-trans-configured substitution pattern at the cyclopentanone core. Comparison of the sample with the commercial standard (±)-jasmonic acid and analytical reference data from [41] confirmed this interpretation. The absolute configuration could not be determined experimentally, yet, previous reports described (-)-JA as the dominant form produced by the fungus *B. theobromae* [23].

**Fig. 1:**
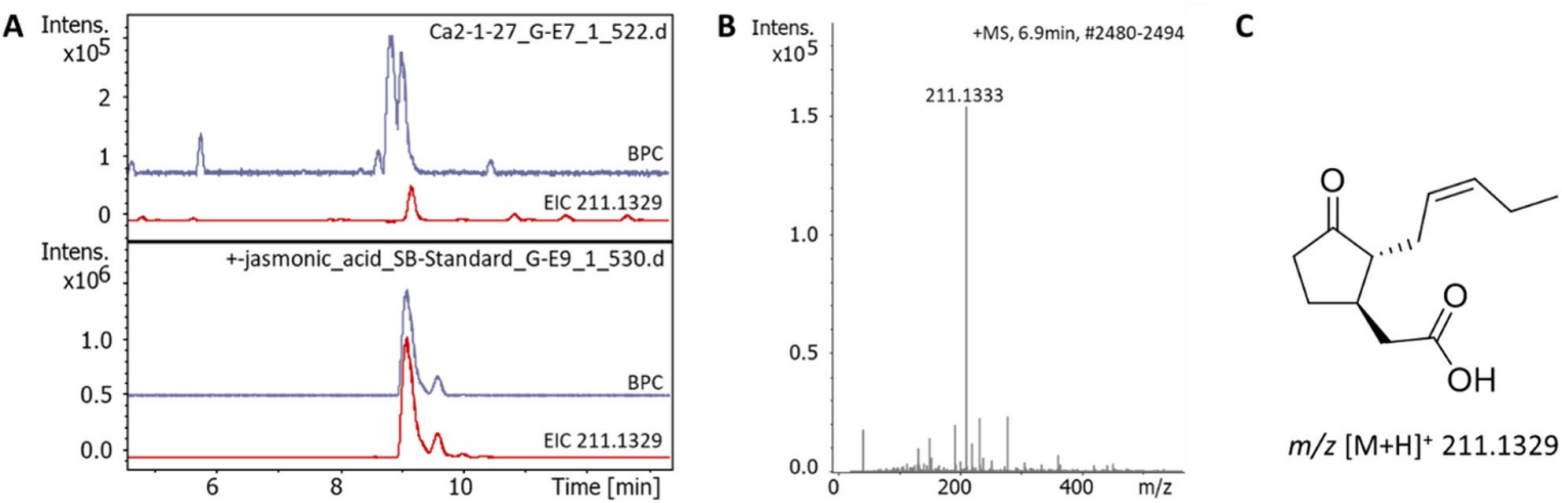
UHPLC-HR-MS analysis of *C. asteris* extract.** A** Chromatograms of extract and JA reference, BPC (blue) and EIC of JA (*m/z* of 211.1329 [M + H]^+^) (red). **B** MS spectrum of JA. **C** Structure of JA and calculated exact mass. HRMS (ESI+) *m/z* calculated for C_12_H_18_O_3_ [M + H]^+^: 211.1329; found: 211.1333

The findings were confirmed by UHPLC-ESI-HR-MS (Fig. 1) and -MS/MS (Suppl. Figure 2) measurements of the respective masses. Taking together the results of the NMR and LC-MS/MS analyses, we could clearly identify the unknown substance in the liquid culture of *C. asteris* as JA (Suppl. Figure 1–7).

### *C. asteris* secretes most of the synthesized JA into the medium

**Fig. 2:**
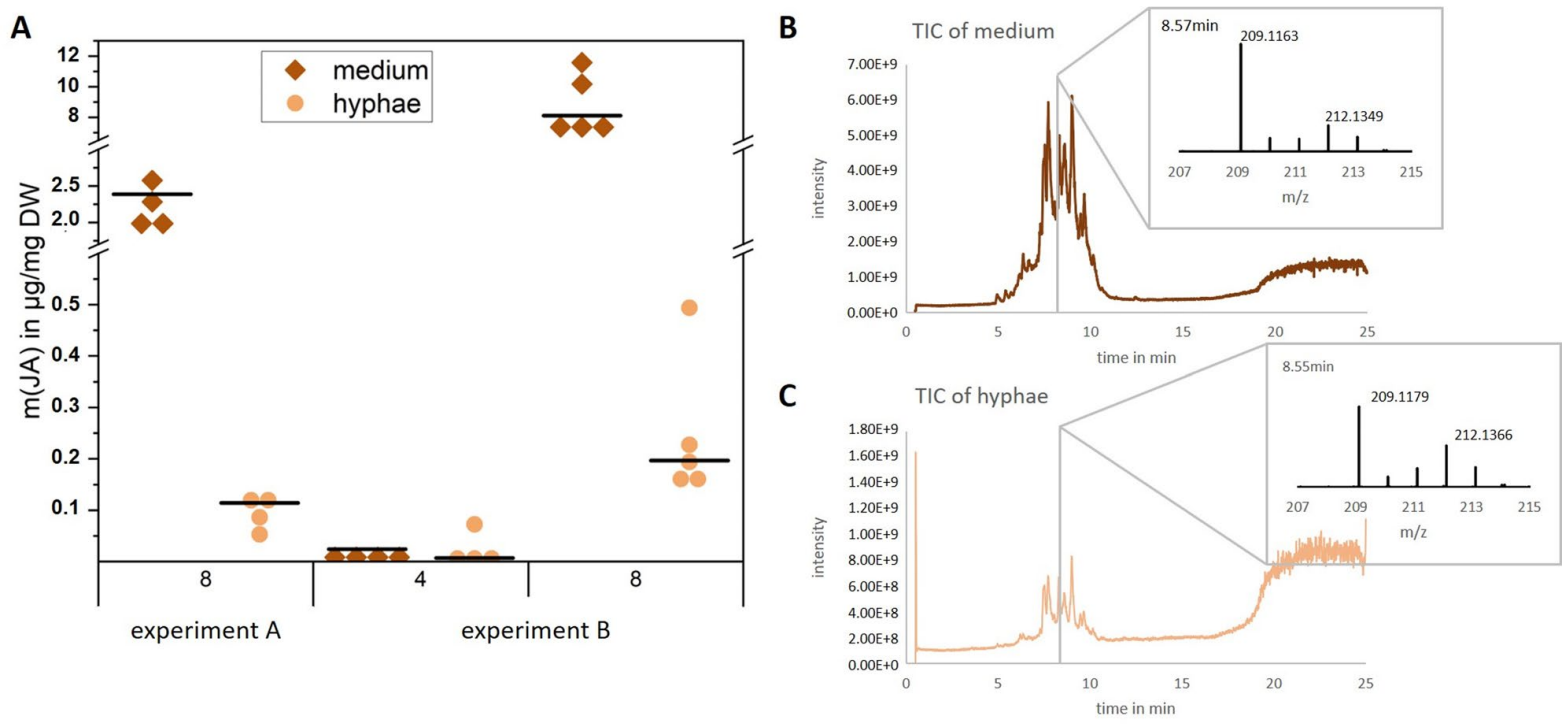
Amount of (±)-JA in medium and hyphae of different *C. asteris* cultures. *C. asteris* was cultivated during two different experiments (A: eight weeks; B: four and eight weeks) under continuous shaking at 23 °C. JA was extracted from medium and hyphae with ethyl acetate, cleaned over an SPE column and measured by LC-MS/MS in negative mode. **A** JA content in medium and hyphae of two different *C. asteris* cultivations. Shown are the median of four biological replicates. **B** TIC with mass spectrum at 8.57 min of a medium sample (supernatant). **C** TIC with mass spectrum at 8.55 min of the same sample, but the hyphae. Indicated are the *m/z* [M-H]^-^ of JA ([M-H]^−^209.12 g/mol) and D_3_-JA ([M-H]^−^212.14 g/mol). DW: dry weight

Besides the culture medium, we also analyzed hyphae of *C. asteris* to clarify if the fungus secretes the synthesized JA or if the JA is only a side product of its own metabolism. Therefore, different fungal in vitro cultures were analyzed by HPLC-MS in negative mode for their JA production after four and eight weeks of cultivation (Fig. 2). The internal standard (±)-D_5_-JA, which was used for JA quantification in the samples, was detectable in each medium and hyphae sample (Fig. 2B **+ **C) as (±)-D_3_-JA with a *m/z* of 212.13 (calculated for (±)-D_3_-JA, C_12_H_14_D_3_O_3_^−^ *m/z* [M-H]^−^ 212.1371). The loss of two deuterium labels during the extraction process and LC-MS can be explained by de-deuteration/re-protonation events at the two acidic α-position in JA [13, 61]. Quantification shows, that most of the biosynthesized JA (*m/z* [M-H]^−^ 209.1183) was secreted into the medium of the *C. asteris* culture, with only very small amounts being detected in the hyphae (Fig. 2A). The relative proportion of JA in the medium increased with cultivation time from 19 to 40% after four weeks to 94–98% after eight weeks.

### The plant precursor LA does not induce JA biosynthesis in *C. asteris*

Until now, no JA biosynthetic pathway has been identified in fungi. In plants, JA is derived from LA. To examine whether *C. asteris* uses a similar pathway, cultures were supplemented with various concentrations of LA dissolved in ethanol, DMSO, or methanol to control for solvent effects. JA levels were measured after four and eight weeks.

**Fig. 3:**
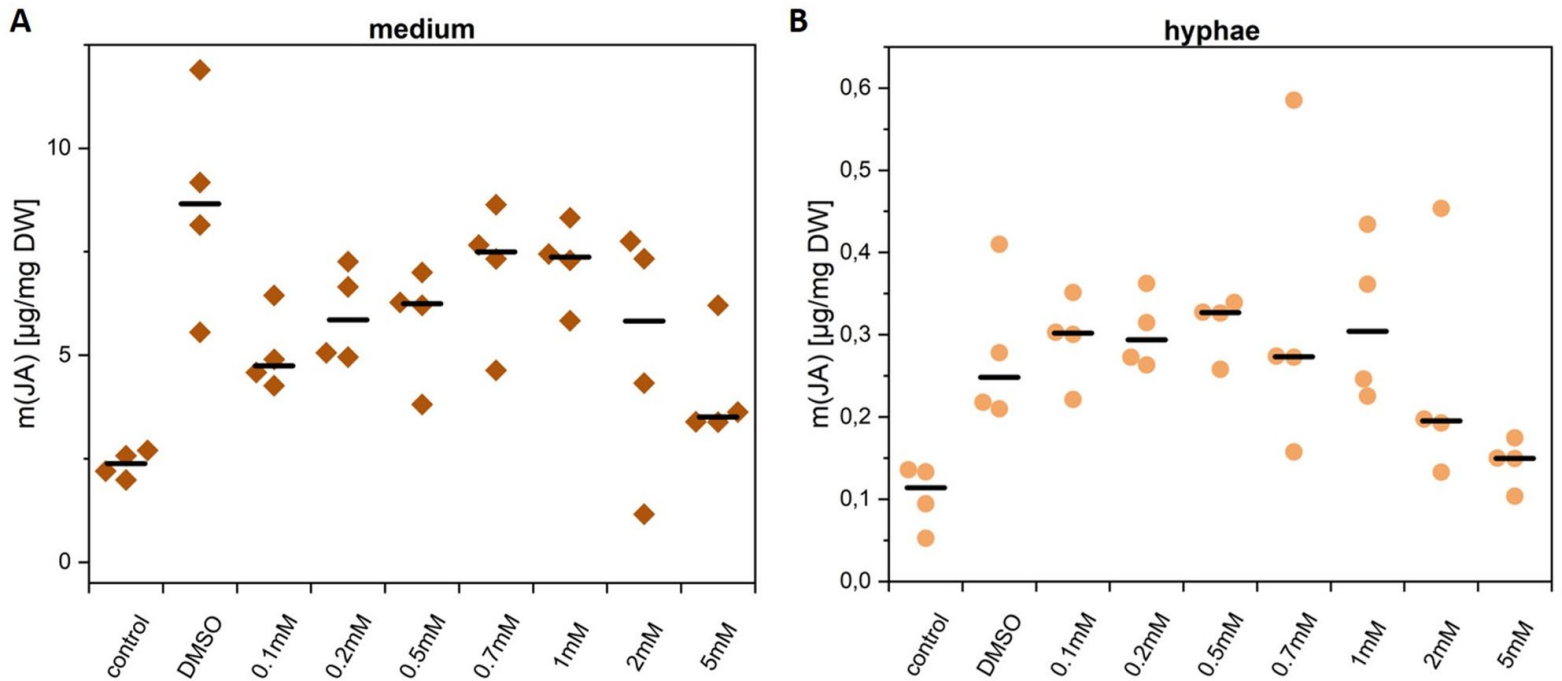
JA amount in *C. asteris* cultures after feeding with LA (DMSO). *C. asteris* was cultivated at 23 °C under continuous shaking, different amounts of LA (0.1 mM to 5 mM, dissolved in DMSO) were added after one week of cultivation. JA content was analyzed after eight weeks by LC-MS/MS. **A** JA amount in the medium. **B** JA amount in the hyphae of *C. asteris*. Shown are the medians of four biological replicates. DW: dry weight

LA dissolved in DMSO appeared to stimulate JA production in both the medium and fungal hyphae (Fig. 3), with the highest induction at 0.7-1 mM LA (medium) and 0.5 mM (hyphae). However, only the DMSO-treated medium showed a significant increase in JA compared to the control (*p* < 0.05). Notably, DMSO alone increased JA 3.6-fold in the medium and 2.2-fold in the hyphae, suggesting that DMSO and not LA induced JA synthesis. Higher LA concentrations even reduced JA levels compared to DMSO alone. Methanol, used at the same low concentration (< 0.1%), had no effect (Suppl. Figure 3).

No consistent LA-dependent effect on JA biosynthesis was observed at either time point. Only one significant difference (between 0.1 mM and 5 mM LA) was found after four weeks. Consistent with previous observations, JA secretion increased over time: 59% after four weeks and 95% after eight weeks. Total JA production rose sharply between week four and eight, with < 1% produced by week four. LA had little to no effect on JA biosynthesis in *C. asteris*. The main inducer was DMSO, even at concentrations below 0.1%.

### Effect of exogenous JA on *C. asteris* fungal biomass

**Fig. 4:**
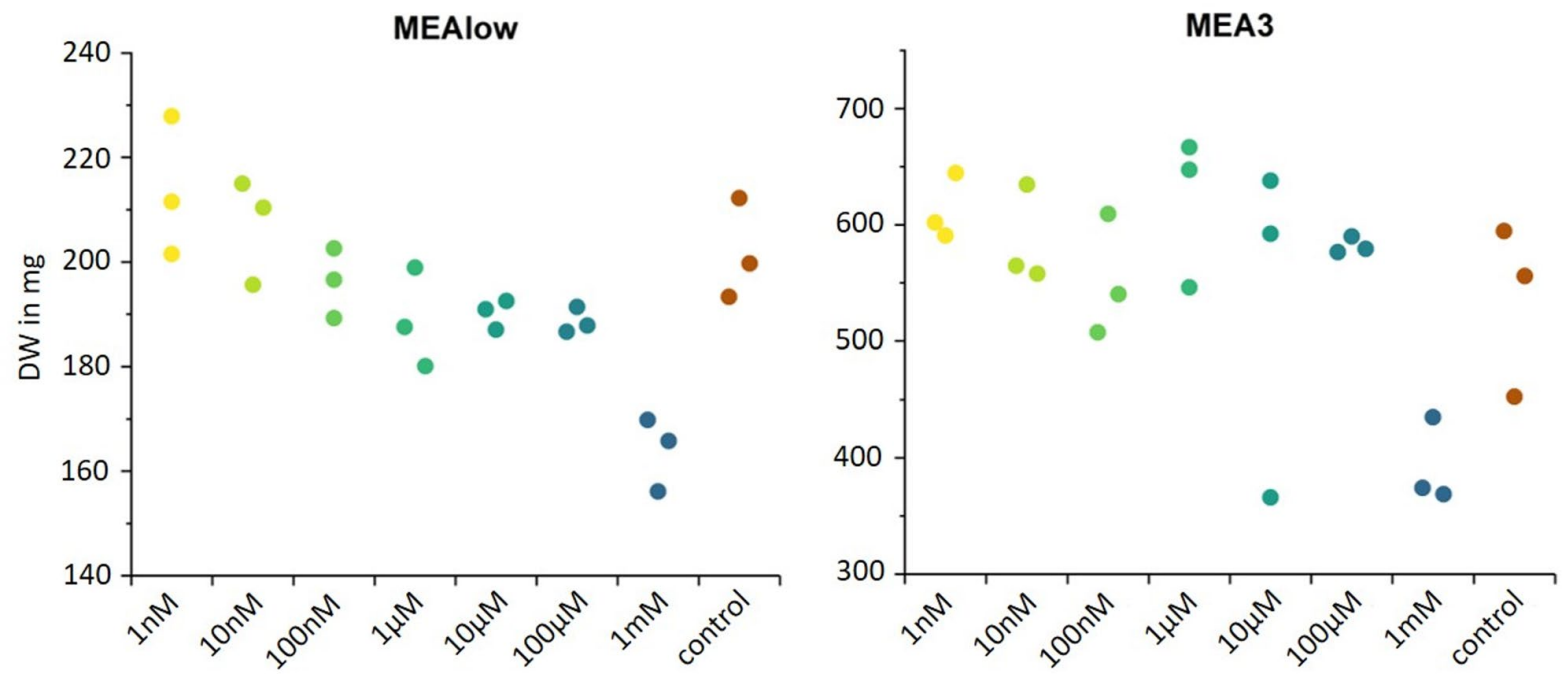
Growth of *C. asteris* in the presence of exogenous JA after four weeks. *C. asteris* was cultivated in minimal medium MEAlow and complete medium MEA3 as shaking cultures (triplicates) for four weeks at 23 °C. Exogenous (±)-JA (1 nM to 1 mM) was added after one week of cultivation. Biomass was determined after four weeks by dry weight (DW)

The influence of exogenous JA on one week-old *C. asteris* cultures has already been investigated [28]. We analyzed the effect after four weeks of cultivation in minimal medium MEAlow and complete medium MEA3 to shed light on the influence of JA on long-term *C. asteris* cultures (Fig. 4). Overall, *C. asteris* grew much better in the complete medium MEA3 than in the minimal medium MEAlow. The biomass of *C. asteris* in the minimal medium MEAlow decreased with an increasing concentration of exogenous (±)-JA. Within the complete medium MEA3, JA had no influence on the biomass production of *C. asteris*, except at the highest JA concentration of 1 mM, where a clear inhibition in biomass formation was evident.

### How is JA synthesized in *C. asteris*?

**Fig. 5:**
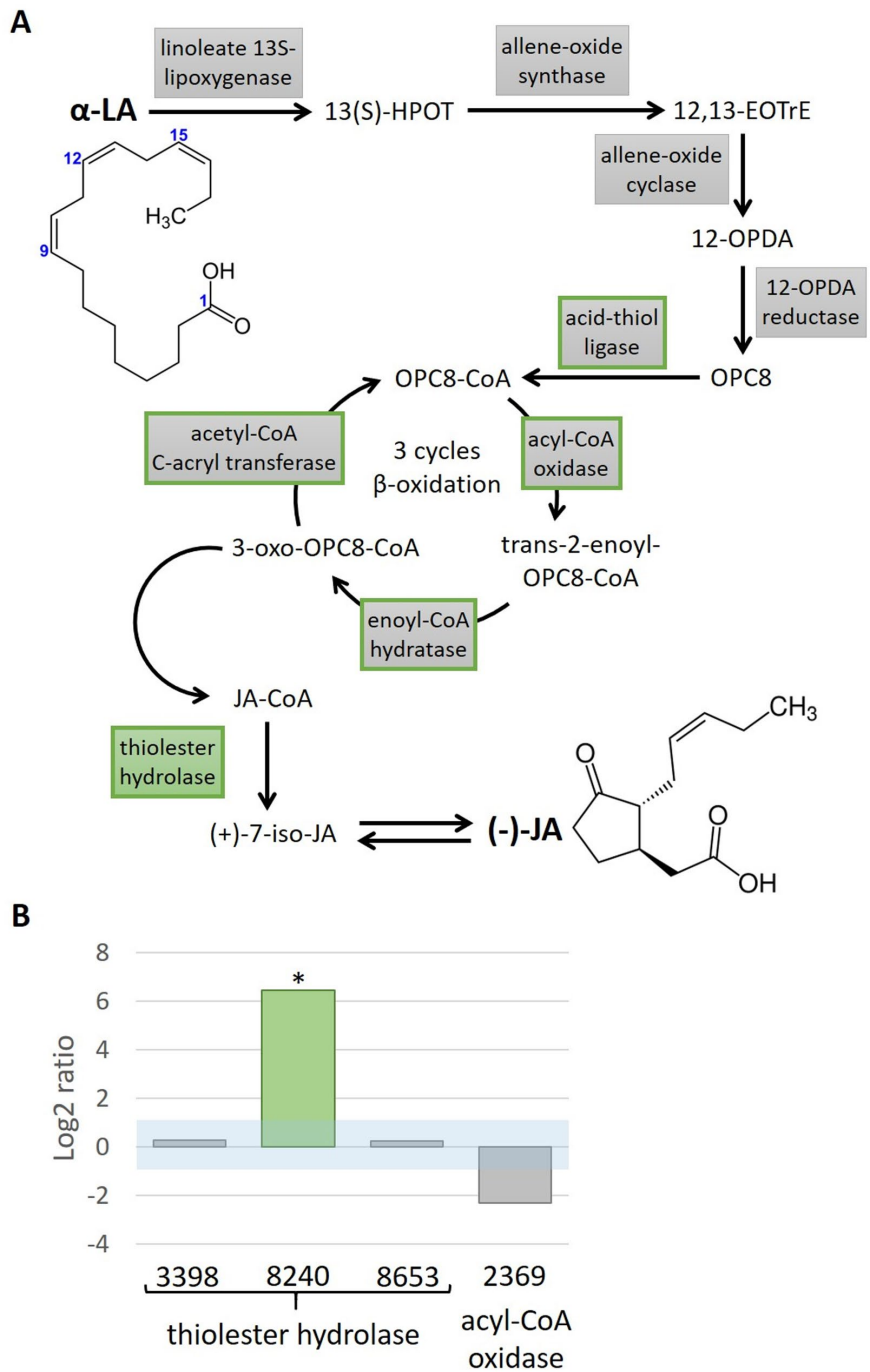
Putative JA biosynthesis in *C. asteris*. **A** Biosynthetic pathway in plants according to Wasternack and Hause [64]. OPC8-CoA is formed from LA in several steps, which is converted into JA-CoA by three cycles of β-oxidation. This is converted in a final step into (+)-7-iso-JA, which then rearranges into the isomer (-)-JA. Enzymes, for which gene candidates were found in the genome of *C. asteris*, are framed in green. 13(S)-HPOT: (9Z,11E,15Z)-(13 S)-hydroperoxyoctadeca-9,11,15-trienoate; 12,13-EOTrE: (9Z,15Z)-(13 S)-12,13-epoxyoctadeca-9,11,15-trienoic acid; 12-OPDA: 12-oxophytodienic acid; OPC8: 3-oxo-2-(2-pentenyl)-cyclopentane-1-octanic acid. **B** Expression analysis of four different JA biosynthesis gene candidates (*3398_g*, *8240_g*, *8653_g* and *2369_g*) identified in the *C. asteris* genome. Expression was compared between cultures with high and low JA production titers. The light blue area indicates the log2 ratio with no regulation. Log2 ratios <-1 show a down-regulation, log2 ratios > 1 show an up-regulation. The asterisk indicates significantly regulated genes

The JA biosynthetic pathway in plants starts with LA as a precursor (Fig. 5A). Using the genome information of *C. asteris* [27], we found several putative candidates within the pathway (Fig. 5A, circled in green), even though JA biosynthesis could not be induced by the precursor LA. Genes encoding for enzymes transforming LA to OPC8 were not identified in the automatic annotated genome of *C. asteris*. Genes were only identified for enzymes converting OPC8 into JA, including acid-thiol ligase, acetyl-CoA oxidase, enoyl-CoA hydratase, acetyl-CoA C-acryl transferase and thiolester hydrolase. qPCR with four candidate genes, three encoding putative thiolester hydrolases (3398_g, 8240_g and 8653_g), which are involved in the last step of the putative JA biosynthetic route by hydrolyzing CoA from JA-CoA, and one is encoding a gene in the ß-oxidation itself (2369_g; acyl CoA oxidase). The latter could be part of the catabolism of fatty acids. The expression was calculated for cultures growing eight weeks versus four weeks. The former had the much higher JA content (Fig. 2). One of the putative thiolester hydrolases was strongly and significantly up-regulated in this comparison (Fig. 5B).

### Fungal JA can rescue the JA phenotype in *A. thaliana*

The JA biosynthesis mutant of *A. thaliana aos10-1* shows a JA-deficient phenotype, such as a prolonged inflorescence phase with a postponed flowering and seed development [44]. This is caused by the knockout in the allene oxide synthase gene (*AOS*) whose gene product is the first enzyme in the plant biosynthetic pathway leading to JA [65].

**Fig. 6:**
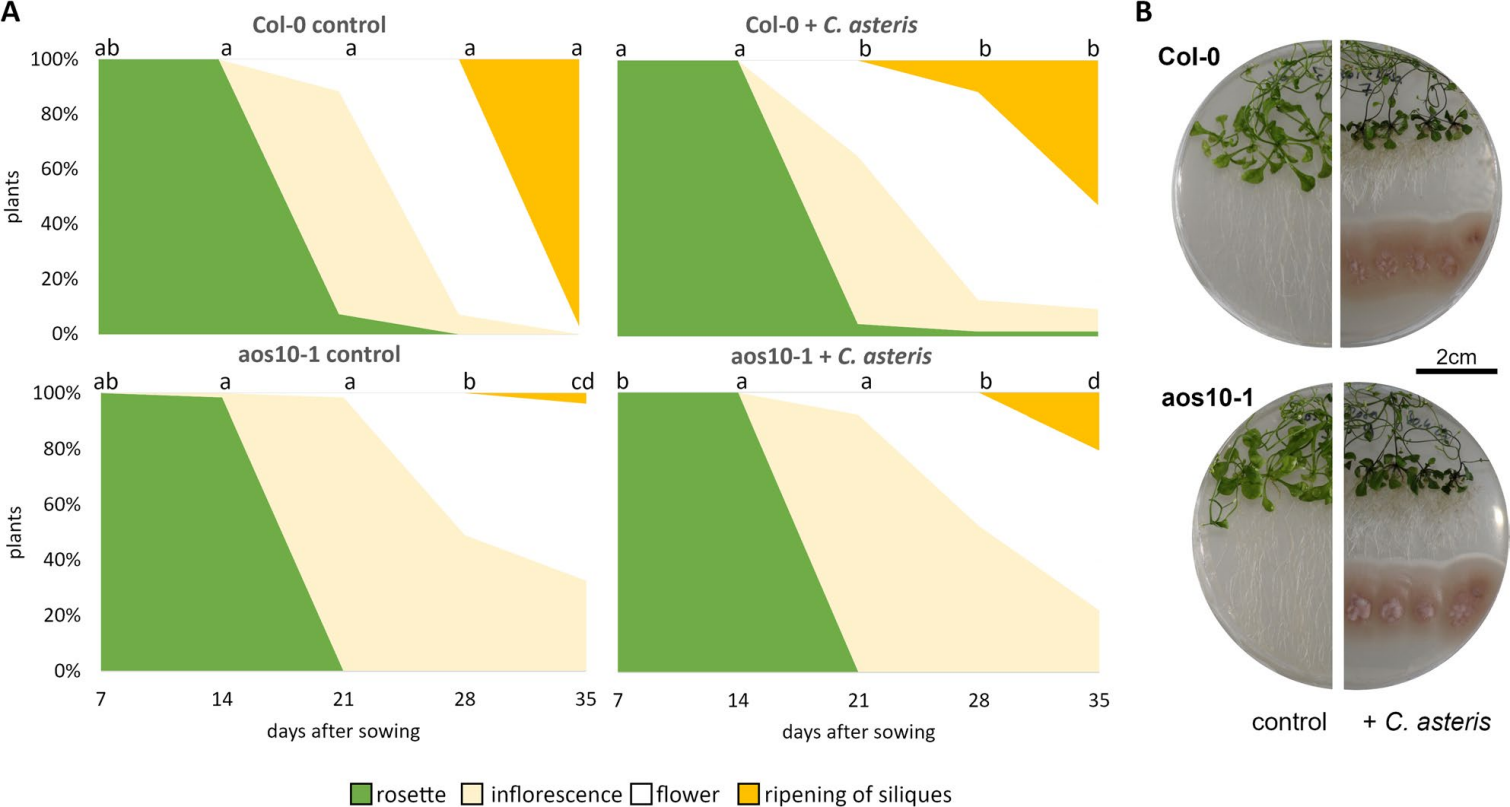
Growth of *A. thaliana* JA biosynthesis mutant *aos10-1* with *C. asteris* after 35 days of cultivation. *A. thaliana* seeds of ecotype Col-0 and mutant *aos10-1* were sown on ½ MS/MEAlow agar and cultivated under long day conditions in a climate chamber. *C. asteris* was put on plates in a distance of 4.5 cm to the seeds after one week of cultivation. **A** Growth stages of *A. thaliana* over a time of 35 days with rosette, inflorescence, flower and seed development. Growth stages between the treatments were statistically analyzed for each time point (*p* < 0.05, Kruskal-Wallis ANOVA, Dunn’s test). **B** Plates after 35 days of cultivation

The *aos10-1* mutant showed a delayed flowering and seed development compared to the ecotype Col-0 (Fig. 6A). *C. asteris* induced flowering and seed development in the ecotype Col-0 as well as in the *aos10-1* mutants, even if the number of plants in the respective growth stages is much higher in the ecotype Col-0 than in the *aos10-1* mutant. All plants showed the typical phenotype, already described before in Jahn et al., [26], namely all *A. thaliana* plants developed in the presence of the fungus *C. asteris* a shorter main root that was compensated by a massive increase in lateral root growth (Fig. 6B).

In addition to the JA biosynthesis-deficient mutant *aos*10-1 of *A. thaliana*, we also analyzed the co-cultivation of the JA-signaling mutant *jar1-1* with *C. asteris* (Suppl. Figure 4). Both ecotype and mutant showed the known phenotype with a shortened main root and a massive lateral root growth. *C. asteris* led to an earlier flowering in the plants.

## Discussion

The phytohormone JA is usually synthesized by plants to regulate abiotic and biotic stress responses, but also growth and development [21]. Until now, only a few fungi are known to produce jasmonates such as *L. theobromae*, *F. fujikuroi* [12], or *F. oxysporum* [40], which are phytopathogens [1] that use jasmonates predominantly to reduce the plant stress response during infection [14] and to induce their own sporulation and mycotoxin production [8]. Based on our findings, *C. asteris* is the first plant endophyte known so far to be able to synthesize JA.

Most of the fungal JA was secreted by *C. asteris* into the medium over time (Fig. 2), indicating a function within the host plant and not within the endophyte itself. Only small amounts were retained in its hyphae. Two out of three strains of *L. theobromae* secreted only small amounts of JA into the medium (0.2 mg/l), whereas the third strain secreted high amounts (200 mg/l) [29]. Secretion of JA into the medium supports the idea that fungal JA is used by the fungus to inhibit the immune response of the host plant and thus to facilitate the infection of the plant by the fungus [14]. JA together with salicylic acid plays a crucial role in plant immunity [15, 17]. They control defense mechanisms for different types of microbial attacks: JA is usually induced by herbivores and necrotrophic pathogens, whereas salicylic acid is induced by biotrophic and hemi-biotrophic microorganisms [17]. JA and salicylic acid pathways are generally antagonistic. An increased resistance to necrotrophs is often correlated with an increased susceptibility against biotrophs [18]. If *C. asteris* increases JA levels in the plant, the door opens for colonization by biotrophic fungi like itself and supports the plant by herbivory and necrotrophy.

The *A. thaliana* plants showed the typical IAA-related phenotype of altered root systems, already known from different *A. thaliana* ecotype and mutant plants [26, 28]. Co-cultivation of *C. asteris* with *A. thaliana* JA-mutants showed that fungal JA can partially rescue the JA-deficient phenotype of the *aos10-1* mutant (Fig. 6). *C. asteris* induced flowering and seed development in *A. thaliana*, not only in the mutant, but also in the ecotype. This might be due to the presence of additional phytohormones in the medium produced by *C. asteris*. Another possibility to alter plant JA biosynthesis would have been to use JA synthesis inhibitors. However, our decision for the mutants was based on the possibility that the plant JA biosynthesis inhibitor could also inhibit fungal JA biosynthesis. Once a fungal biosynthetic pathway has been established such experiments are valuable options to validate our findings with wildtype and mutant strains of *A. thaliana*. The co-cultivation of the JA signaling mutant *jar1-1* with *C. asteris* did not change the phenotype compared to the ecotype Col-0. We could already show that *C. asteris* produces IAA, which is mainly responsible for the typical IAA phenotype of the plants [26, 28]. Several endophytes are known to produce phytohormones like auxins, gibberellins or ethylene to adapt the plant environment and to support the plant like *Aspergillus ustus* [50] or *Bacillus megaterium* [36]. For IAA, it was shown that uptake of the compound is realized by the uptake facilitators AUX/LAX [26]. The inward transport of JA across the plasma membrane has been attributed to a set of ABC transporters, namely JAT3 and JAT4 of *A. thaliana* (reviewed in Anfang and Shani [2], which makes its interaction with JA-producing endophytes feasible. Notably, it was demonstrated that resistance to *Botrytis cinerea* is dependent on an intact JA transport in tomato [56]. Besides fungal JA, it is also important to consider JA derivatives, such as JA-CoA or methylated JA, which exhibit similar effects on plants (reviewed in [38]). However, we did not detect any of these derivatives in the fungal extracts, likely as a consequence of the already low levels of the parent compound JA present. As a result, we cannot determine whether or how these derivatives potentially contribute to the rescue of the JA-deficient phenotype in *A. thaliana* mutants by *C. asteris*. This is critical, as these derivatives may also influence plant responses, and their potential impact cannot be ruled out. Yet, it is particularly difficult to distinguish between the effects of JA and the individual derivatives, since fungal JA could be channeled into the manifold JA-derivatizing pathways of *A. thaliana*, leading to plant-produced JA congeners, which can exhibit the same effect as identical derivatives putatively produced by the fungus itself. To validate our findings that fungal JA produced by *C. asteris* alone can rescue JA-deficient *A. thaliana* plants, complimentary experiments would be highly beneficial.

Exogenous (±)-JA affected *C. asteris* differently depending on the cultivation medium: In the minimal medium MEAlow, it caused a decrease in biomass. In the complete medium MEA3, there was no significant effect of JA on the biomass of *C. asteris* (Fig. 4). In the minimal medium MEAlow, the biomass decrease was caused by JA toxicity at higher concentrations. The bacteria *Streptomyces* and *Streptacidiphilus* show a similar phenomenon at higher exogenous JA concentrations: they can detoxify exogenous JA by conjugation with different amino acids up to a specific concentration, but above they are no longer able to do so and reduced growth [59]. One-week-old *C. asteris* cultures showed no JA-induced change in biomass in MEAlow [28], possibly due to the short cultivation time and the rather low JA concentrations measured in these experiments. In comparison to the MEAlow medium, the fungus in MEA3 medium exhibited significantly higher biomass production. Minimal media are defined as media with limited nutrient content, enabling microbial survival but hindering optimal growth. Microorganisms exhibit increased sensitivity to environmental fluctuations, and complete media provide ample nutrients to facilitate their growth, enabling them to withstand elevated JA concentrations due to the greater nutrient availability compared to minimal media. The addition of (±)-JA increases the production of astin C in *C. asteris* [28]. This enhanced astin C biosynthesis is thought to support the host plant during stress responses, as the plant-derived JA signals the endophytic fungus to initiate astin C production. A similar phenomenon is known from *Epichloe* endophytes, which produce alkaloids and non-alkaloids to protect the host grasses (reviewed in Song et al., [55]. Additionally, *Epichloe* uses JA to support the immune response in grasses during herbivory by chewing insects [4]. Many endophytes support the survival of their host plants by promoting growth and fitness [6, 22, 49, 62]. *Epichloe typhina* promotes the photosynthesis and growth of its host plant *Dactylis glomerata* [49]; dark septate endophytes support maize during cadmium stress [62]; and *Paecilomyces* sp. increase resistance to combined abiotic stresses in *Glycine max* [6]. Endophytes have a huge ability to help their host plants during difficult conditions like drought, salinity, or heat [6, 20, 31]. They are also effective in increasing the resistance of their host plants to biotic stresses like pathogen attack or herbivory [4, 48]. *C. asteris* is another endophyte in the row of fungi supporting their host plants.

The precursor of plant JA biosynthesis LA was added to *C. asteris* cultures to induce fungal JA biosynthesis, but did not lead to a significant induction of JA biosynthesis in the fungus (Fig. 3, Suppl. Figure 3). It was reviewed that fungal oxylipins could also derive from oleic and linoleic acids as precursors based on protein sequences and annotation of functional groups [5]. Therefore, we cannot rule out that in *C. asteris* a different precursor and thus pathway to JA is active. Limited cellular uptake of LA might be another reason. *Lasiodiplodia theobromae* uses LA to synthesize JA over OPDA [39, 57]. Additional feeding experiments with other fungi support the hypothesis that fungal JA is synthesized in a similar way as in plants, probably using the same enzymes as in plants (reviewed in Eng et al., [12]. We could not detect increased amounts of JA in *C. asteris*, independent from the LA concentration in the culture medium (Suppl. Figure 3). LA is not only the precursor of the JA biosynthesis; it is also an essential part of plant membranes [47]. Therefore, *C. asteris* should be cultivated in medium containing heavy labelled precursors and intermediates to elucidate JA biosynthesis. Interestingly, the solvent DMSO alone led to an increase in JA concentration (Fig. 3). There is evidence that DMSO affects the organization and properties of bilayer lipid membranes [16], even though it is used as ‘mild’ solvent in many applications. As such, it may not cause harm, but facilitate the accessibility for the enzymes that liberate the JA precursors from the respective membrane. Although this is pure speculation in the case of *C. asteris*, it was shown for model membranes that at low concentrations, DMSO induces membrane thinning and increases fluidity, while at higher concentrations, individual lipid molecules were desorbed from the membrane and ultimately the membranes disintegrated [19].

Although, we could not identify LA as a precursor of the JA biosynthesis in *C. asteris*, we analyzed the presence and potential roles of candidate genes in the fungus known for plant JA biosynthesis. Genes encoding all enzymes involved in the conversion of OPC8 to JA were identified in the automated annotation of the *C. asteris* genome (Fig. 5A). In contrast, genes encoding the enzymes required to convert the plant precursor LA into OPC8 were absent, which may explain our observations, that exogenous LA did not enhance JA production (Suppl. Figure 3). The strong up-regulation of a gene encoding a thiolester hydrolase in *C. asteris* cultures with high JA levels (Fig. 5B) indicated that at least the final step from JA-CoA to JA likely takes place in the fungus *C. asteris*. Other fungi, such as *F. oxysporum* or *L. theobromae* also produce (+)-7-iso-JA [12, 43] before it is converted to JA. Other JA intermediates known from plant biosynthesis have already been identified in fungi, such as OPDA or 13(S)-HPOT [43], suggesting that JA biosynthesis is very similar at the chemical level between plants and fungi. Since fungi do not have chloroplasts, where the plant JA biosynthesis starts, it is assumed that the biosynthesis starts in the fungal cytosol [12]. Fungal JA seems to play a crucial role in the interaction between plants and fungi, since it was only identified in fungi associated with plants [12].

## Conclusion

Further experiments are necessary to analyze deeper how *C. asteris* synthesizes JA. Feeding experiments with heavy labelled JA precursors and intermediates from plant JA biosynthesis would shed light on this pathway. This can also help to understand the role of JA between the endophyte and its hosts. Once a JA biosynthesis inhibitor specific to fungal JA biosynthesis is identified, it could be used in combination with JA-deficient *A. thaliana* mutants and *C. asteris*. This approach would allow to clearly demonstrate that the rescue of the JA-deficient phenotype is specifically due to fungal JA and not influenced by other substances produced by the fungus. However, since we currently do not know at which step a potential inhibitor would act in the fungal biosynthetic pathway, further research is required to identify and develop such a specific inhibitor.

## Supplementary Information

Below is the link to the electronic supplementary material.

Supplementary Material 1

## Data Availability

The data are available in the results and supplement or otherwise available upon request from the corresponding author.
