# Supplementary material for: The plant endophytic fungus Cyanodermella asteris produces the phytohormone jasmonic acid
Source: Fungal Biol Biotechnol. 2026 Apr 3;13:4. doi: 10.1186/s40694-026-00210-6 (PMC13063583; doi:10.1186/s40694-026-00210-6)
Supplement: Supplementary file 1 — Supplementary Material 1 [file 40694_2026_210_MOESM1_ESM.docx]

## Supplementary material

**Suppl. Table 1: Primers with their sequences** used to identify possible JA biosynthesis genes as well as the fungal reference genes.

| **primer** | **gene** | **sequence (5‘ to 3‘)** |
| --- | --- | --- |
| qCya3398_F1 | 3398_g | GGCAAGGCTGAGGACACGA |
| qCya3398_R1 | 3398_g | ATGACTTCCCCTCGCAGCCA |
| qCya8240_F1 | 8240_g | CAAAGTGCGCTGGGATGCAA |
| qCya8240_R1 | 8240_g | TTCTGGATCGCTCCTTCCGC |
| qCya8653_F1 | 8653_g | GCGATTTCGAGAGGCTTGGG |
| qCya8653_R1 | 8653_g | ATCCACTCGGCAACCTCGTG |
| qCya2369_F1 | 2369_g | GATTGGCAGCTCGACAGCAG |
| qCya2369_R1 | 2369_g | CCTCAGAGTCGGGGTAAGGG |
| qCyagACT_1,317 F | 6734_g (gACT) | GGTATCTCTGACCGCATGCA |
| qCyagACT_1,456 R | 6734_g (gACT) | TGCTGGAAGGTCGAGAGAGA |
| qCyaTEF1b_796 F | 1700_g (EF1b) | GACATGGCAGAGCTCGAGAA |
| qCyaTEF1b_983 R | 1700_g (EF1b) | GACTGAACGTGGTCCTCGTC |
| qCyaRPB2_3,496_F1 | 6062_g (RPB2) | CTGCGTTTCGGAGAGATGGA |
| qCyaRPB2_3,646_R1 | 6062_g (RPB2) | TCTCCTTGAGCTTGACCACG |


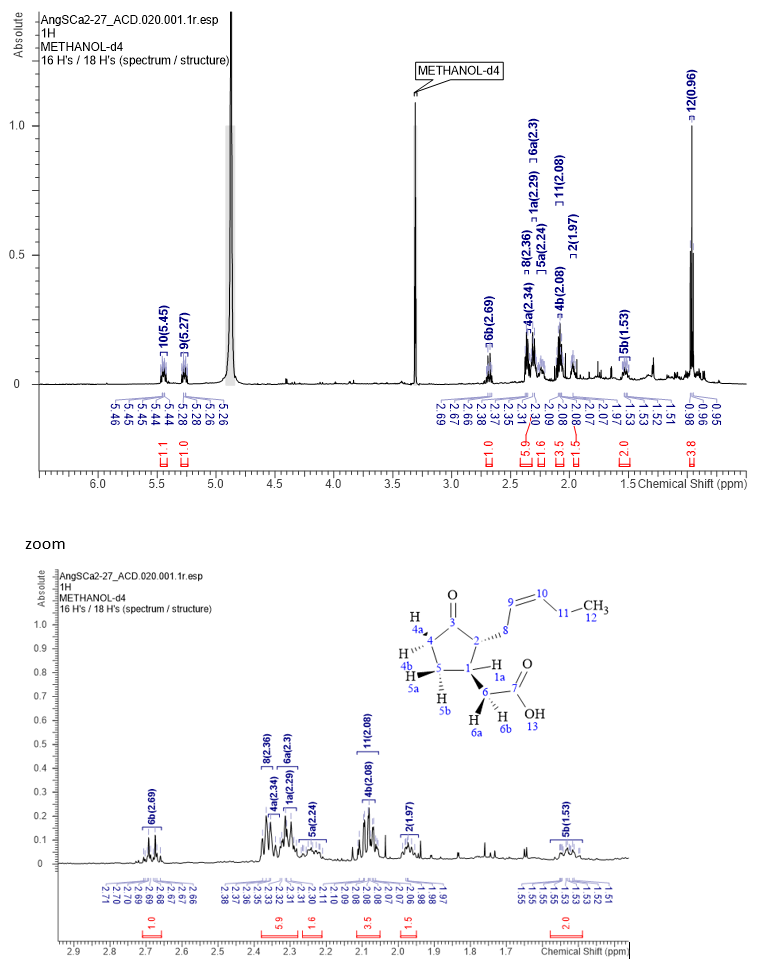


**Suppl. Figure 1: ^1^H NMR spectrum of JA in MeOD at 600 MHz.** Top: full range, bottom: zoom. Chemical shifts for overlapping multiplets were determined from HMBC and HSQC spectra. Stereochemical configuration of protons 4a/b, 5a/b and 6a/b could not be assigned unambiguously.

^1^H NMR spectrum (methanol-d_4_, 600 MHz) *δ* = 5.45 (1H, dtt, *J* = 10.9, 7.2, 1.5 Hz, H-10), 5.27 (1H, dtt, *J* = 10.9, 7.5, 1.5 Hz, H-9), 2.69 (1H, m, H-6b), 2.35 - 2.38 (1H, m, H-8), 2.34 (1H, m, H-4a), 2.28 - 2.32 (1H, m, H-1a), 2.28 - 2.34 (1H, m, H-6a), 2.20 - 2.28 (1H, m, H-5a), 2.06 - 2.10 (1H, m, H-4b), 2.06 - 2.11 (2H, m, H-11), 1.94 - 1.99 (1H, m, H-2), 1.49 - 1.58 (1H, m, H-5b), 0.96 (3H, t, *J* = 7.5 Hz, H-12);.


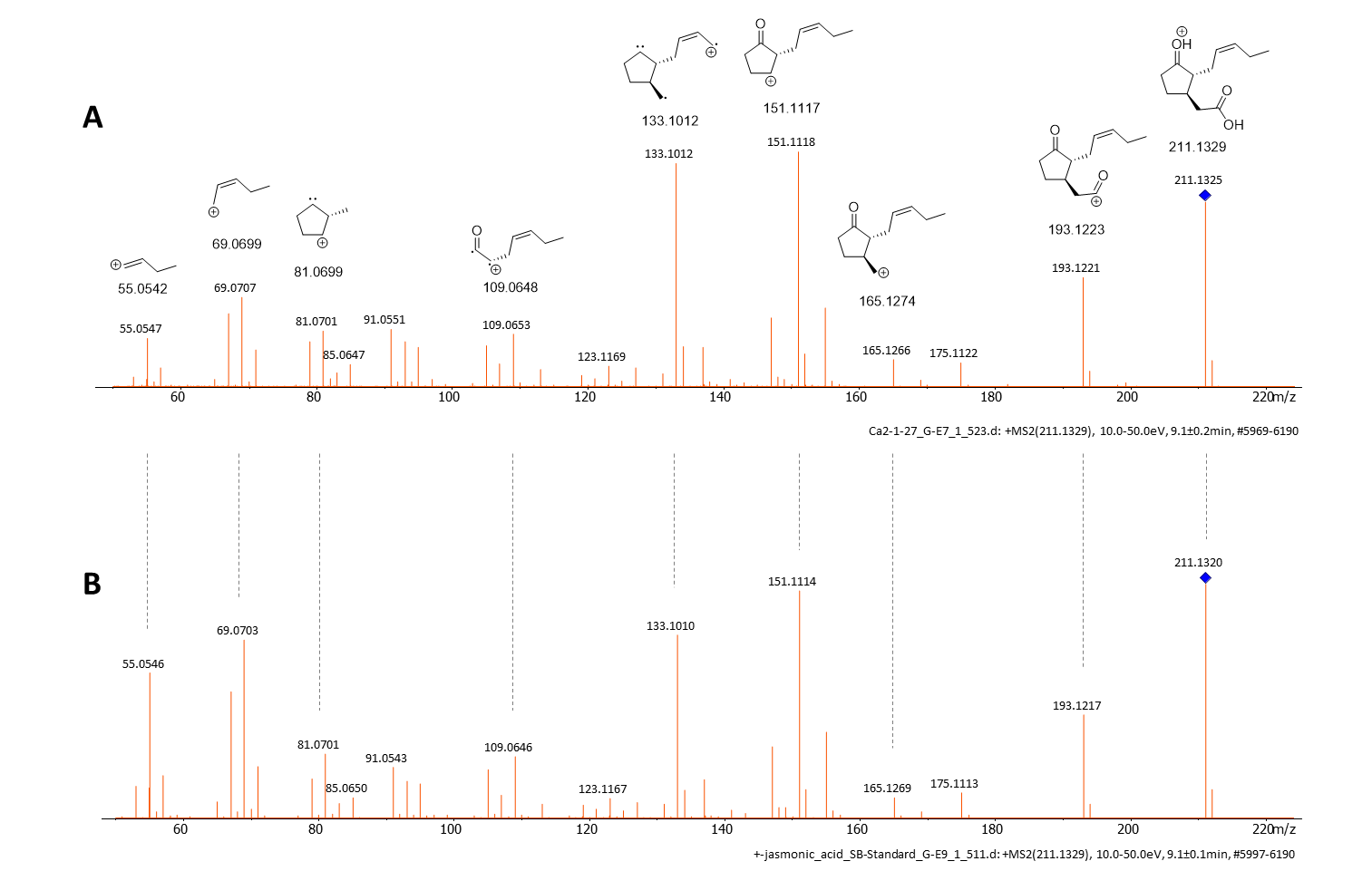


**Suppl. Figure 2:** **MS/MS Fragmentation pattern of JA** **A.** JA found in fraction of *C. asteris* extract. **B.** Commercial standard (±)-JA. Both recorded by UHPLC-ESI-HR-MS/MS in positive mode.

**Suppl. Figure 3: ^13^C NMR spectrum of JA in MeOD at 150 MHz.** ^13^C NMR spectrum (methanol-d_4_, 150 MHz) *δ* = 222.0 (C-3), 176.2 (C-7), 135.0 (C-10), 126.4 (C-9), 55.2 (C-2), 39.9 (C-6), 39.3 (C-1), 38.8 (C-4), 28.3 (C-5), 26.3 (C-8), 21.7 (C-11), 14.6 (C-12).

**Suppl. Figure 4:** DEPT-135 spectrum of JA in MeOD at 150 MHz.


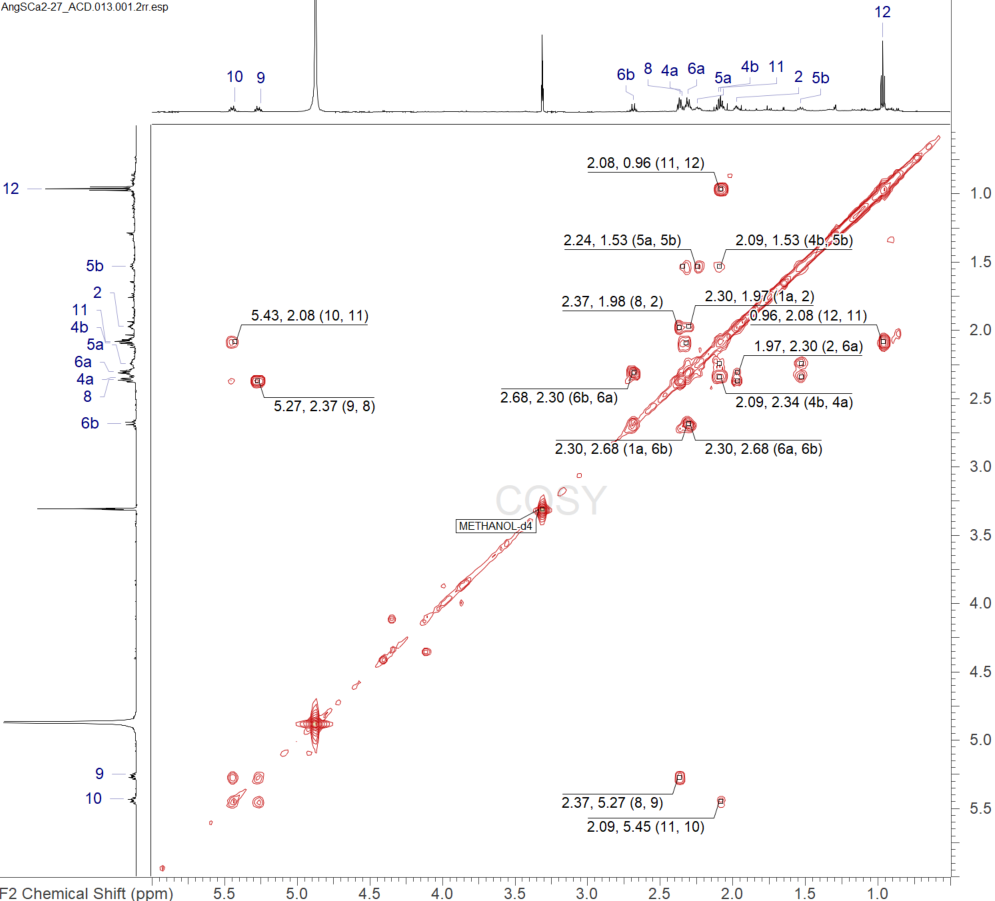


**Suppl. Figure 5:** ^1^H-^1^H-COSY spectrum of JA in MeOD.


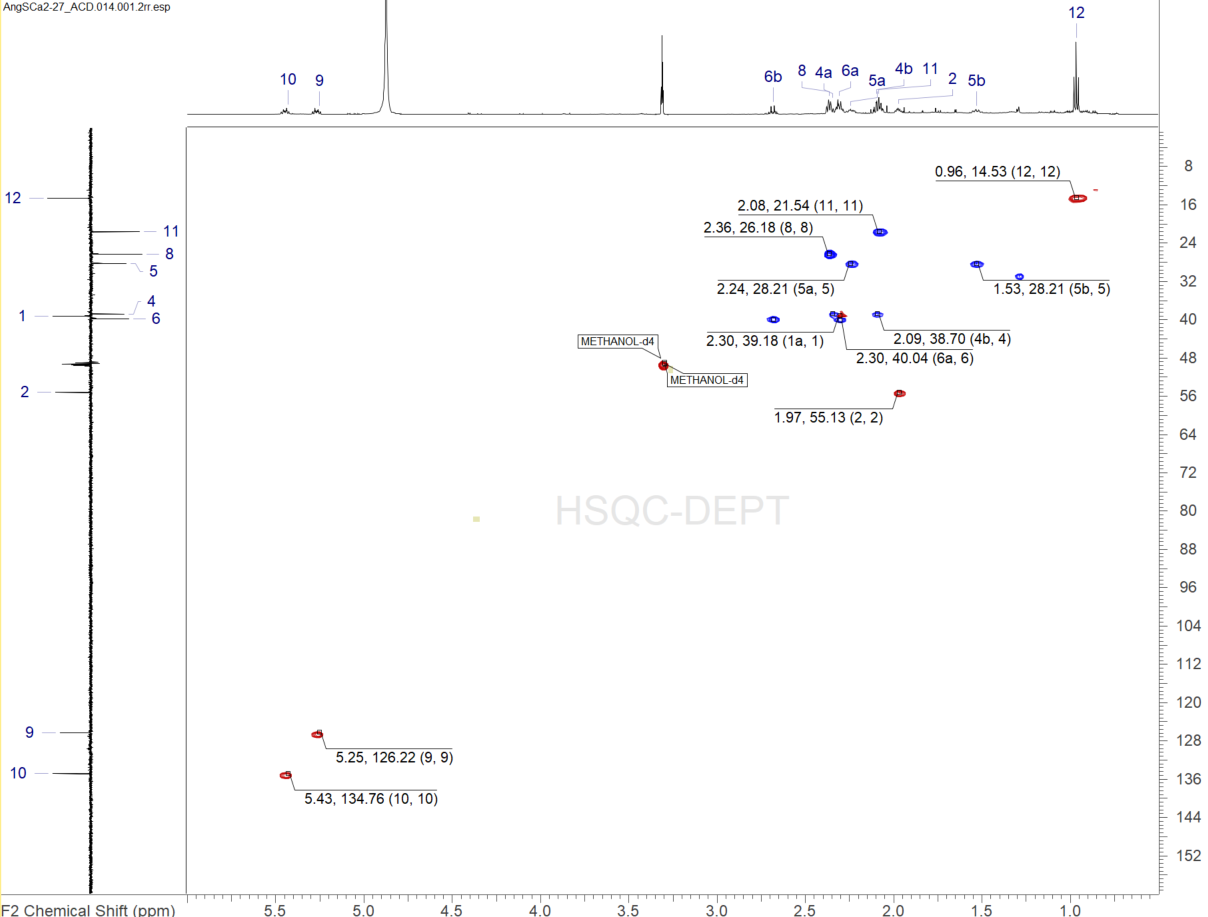


**Suppl. Figure 6:** ^1^H-^13^C HSQC spectrum of JA in MeOD. F1 projection shows the DEPT-135 spectrum.


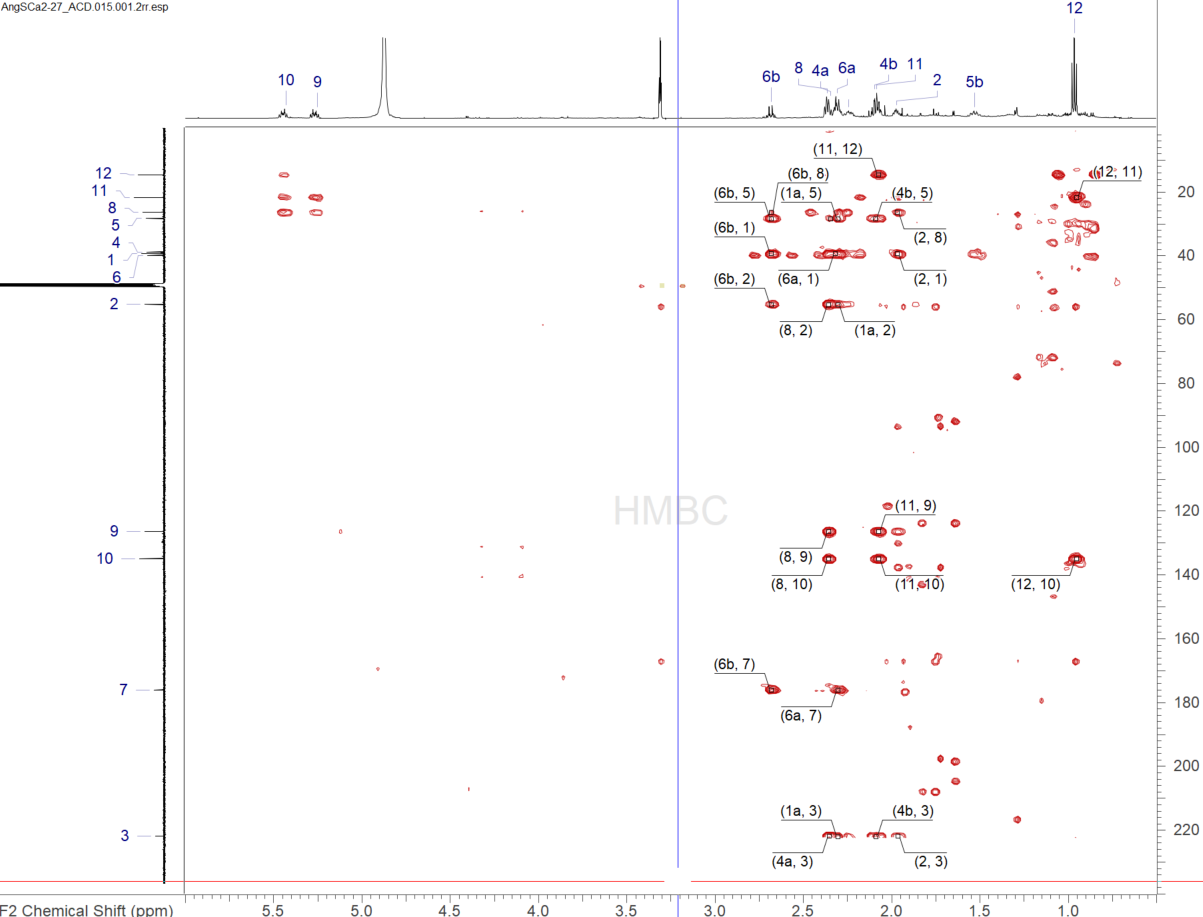


**Suppl. Figure 7:** ^1^H-^13^C HMBC spectrum of JA in MeOD.


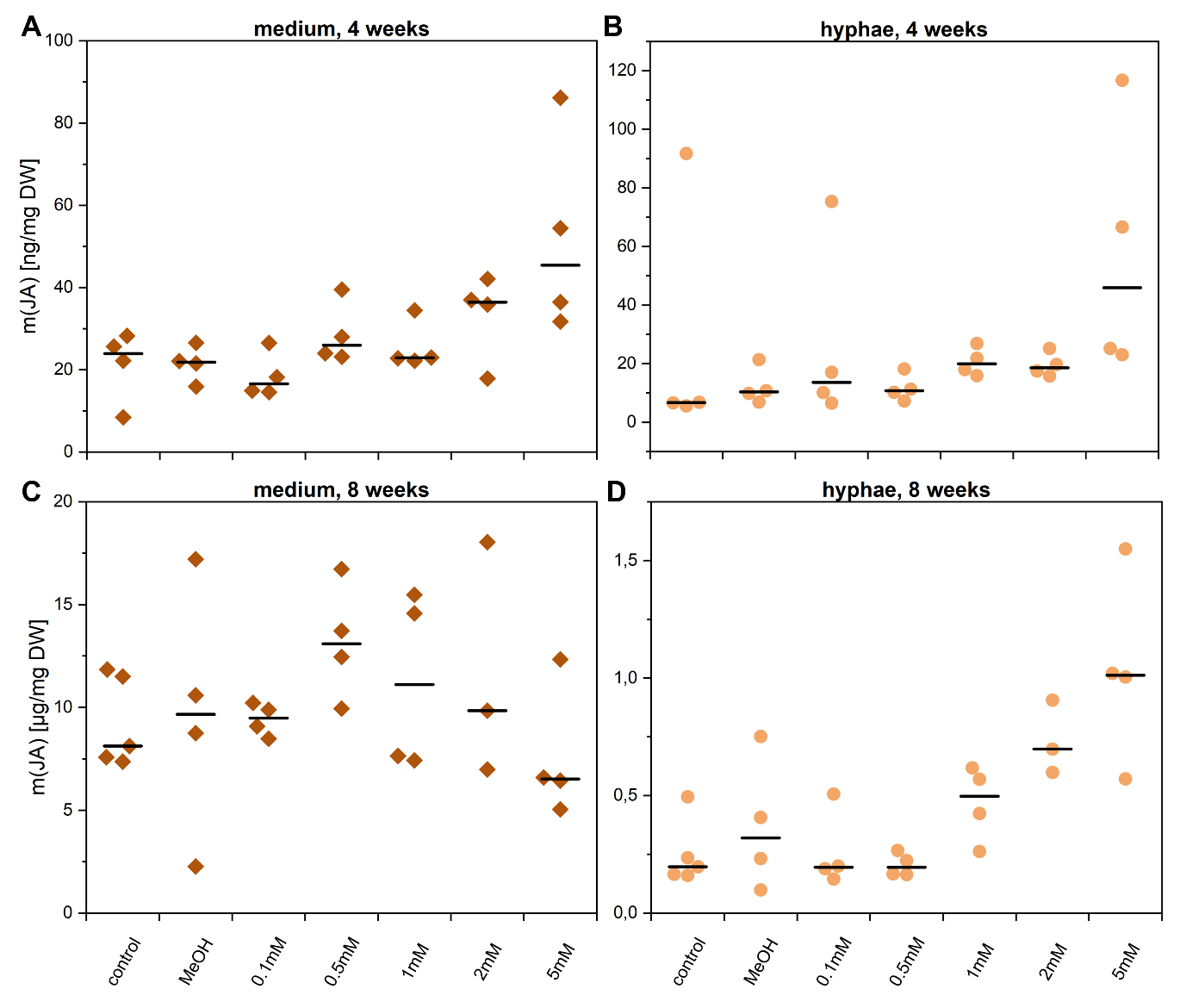


**Suppl. Figure 8: JA amount in *C. asteris* cultures after feeding with LA (MeOH).** *C. asteris* was cultivated at 23°C under continuous shaking, different amounts of LA (0.1 mM to 5mM, dissolved in MeOH) were added after one week of cultivation. JA content was analyzed after four and eight weeks by LC-MS/MS. JA amount in the medium (A, C) and hyphae (B, D) after four (A, B) and eight (C, D) weeks. Shown are the medians of four biological replicates. DW: dry weight.


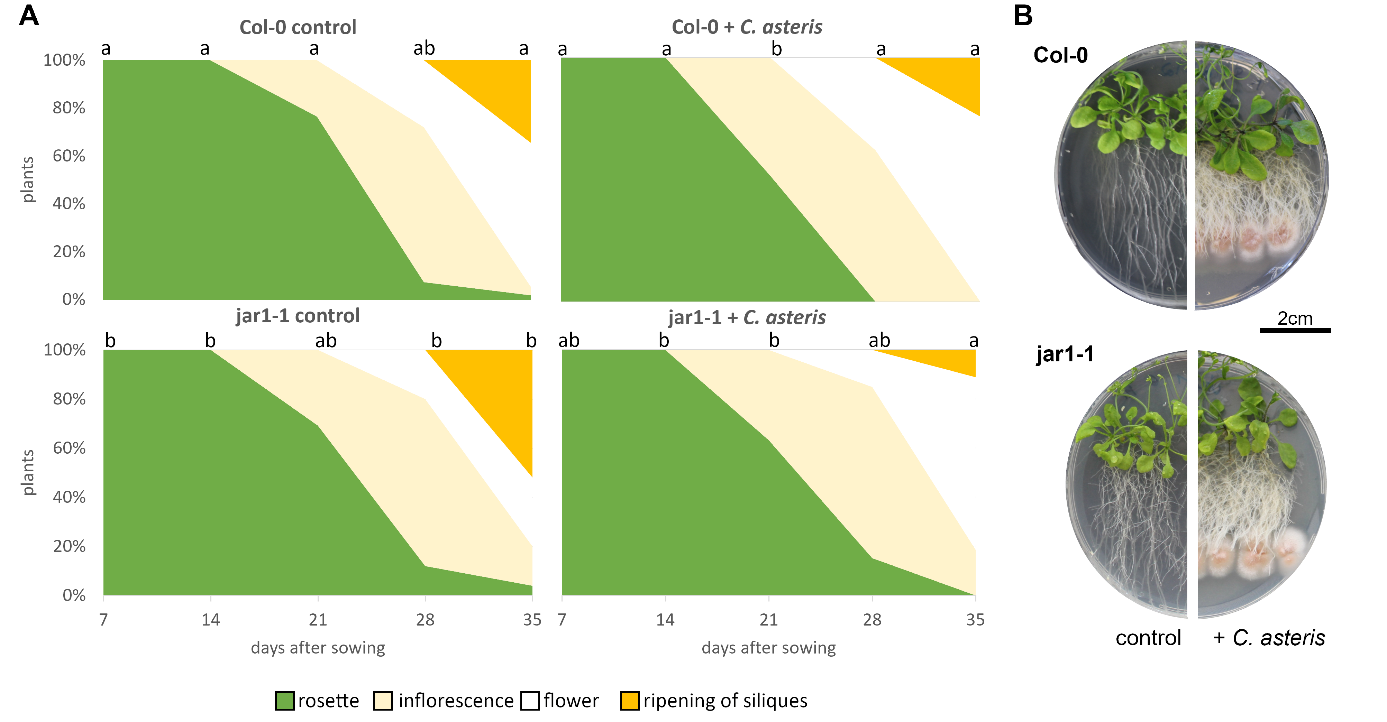


**Suppl. Figure 8:** **Growth of *A. thaliana* JA signaling mutant *jar1-1* with *C. asteris* after 35 days of cultivation.** *A. thaliana* seeds of ecotype Col-0 and mutant *jar1-1* were sown on ½ MS/MEAlow agar and cultivated under long day conditions in a climate chamber. *C. asteris* was put on plates in a distance of 4.5cm to the seeds after one week of cultivation. **A** Growth stages of *A. thaliana* over a time of 35 days with rosette, inflorescence, flower and seed development. Growth stages between the treatments were statistically analyzed for each time point (p<0.05, Kruskal-Wallis ANOVA, Dunn’s test). **B** Plates after 35 days of cultivation.
